# Brexpiprazole augmentation in treatment-resistant obsessive-compulsive disorder: a preliminary retrospective observational study

**DOI:** 10.1097/YIC.0000000000000583

**Published:** 2025-02-06

**Authors:** Luca Giacovelli, Eleonora Piccoli, Paola Landi, Matteo Vismara, Beatrice Benatti, Bernardo Dell'Osso

**Affiliations:** a Department of Biomedical and Clinical Sciences Luigi Sacco, Department of Psychiatry, ASST Fatebenefratelli-Sacco, University of Milan, Milan, Italy; b Department of Psychiatry and Behavioral Sciences, Bipolar Disorders Clinic, Stanford Medical School, Stanford University, Stanford, California, USA; c CRC ‘Aldo Ravelli’ for Neurotechnology & Experimental Brain Therapeutics; d Centro per lo studio dei meccanismi molecolari alla base delle patologie neuro-psico-geriatriche, University of Milan, Milan, Italy

**Keywords:** antipsychotic agents, drug therapy, obsessive-compulsive disorder, off label use

## Abstract

Obsessive-compulsive disorder (OCD) is a chronic illness associated with significant functional impairment. Monotherapy with serotonin reuptake inhibitors (SRIs) often leads to only partial improvement of symptoms. In such cases, a common, well established, treatment approach for most patients is the augmentation of SRI therapy with antipsychotic medications. Brexpiprazole is an atypical antipsychotic agent that acts as a partial agonist of 5-HT1A, D2, and D3 receptors. Purpose of this retrospective observational study was to evaluate the effectiveness and tolerability of brexpiprazole as augmentation to SRIs in patients with treatment-resistant OCD. This preliminary study included a sample of 10 patients diagnosed with treatment-resistant OCD who underwent a 12-week trial of augmentative brexpiprazole, starting at a dose of 1 mg/day, with dosage adjustments based on clinical judgment. Treatment response was assessed through changes in the Yale-Brown Obsessive Compulsive Scale (Y-BOCS) total score from baseline to the end of the 12-week observation period. Adverse events were systematically recorded. Significant improvement was observed after the 12-week period: at the endpoint, seven patients (70%) achieved a ≥25% reduction in Y-BOCS total score compared to baseline, with five of them (50% of the overall sample) showing a more robust clinical response (≥35% reduction). Mild adverse effects, such as sedation and weight gain, were reported by two participants (20% of the overall sample). These findings suggest that brexpiprazole may offer a promising effectiveness and tolerability profile in the management of treatment-resistant OCD.

## Introduction

Obsessive-compulsive disorder (OCD) is a chronic condition characterized by recurrent intrusive thoughts, images, or impulses/urges (obsessions) or repetitive behaviors (compulsions) that individuals feel driven to perform (APA, 2013). The global lifetime prevalence of OCD is estimated at 1.5% in women and 1.0% in men (Cervin, 2023), with illness onset generally occurring during adolescence or early adulthood (Vismara *et al*., 2024). Initial treatment is often guided by symptom severity and patient preferences. For individuals with mild functional impairment or those who prefer non-pharmacological options, guidelines like National Institute for Health and Care Excellence recommend cognitive-behavioral therapy (CBT) with Exposure and Response Prevention (ERP) as the first-line approach. In cases of moderate-to-severe symptoms, a combination of pharmacotherapy and CBT with ERP is typically recommended (NICE, 2013), though pharmacotherapy alone may be considered in certain clinical scenarios (i.e. patient’s preference, incapacity to engage in CBT). Within serotonin reuptake inhibitors (SRIs), selective agents (SSRIs) are generally preferred as first-line pharmacological treatment over tricyclic antidepressants like clomipramine, due to their favorable tolerability profile (Fineberg *et al*., 2015; Skapinakis *et al*., 2016). However, monotherapy often results in only partial symptom relief, with patients experiencing an average 20–40% reduction in symptoms after 12 weeks (Soomro *et al*., 2008; Katzman *et al*., 2014). Clinical studies report treatment with SSRIs or clomipramine to bring significant symptom improvement in only 40–60% of patients. Therefore, adding CBT/ERP or augmenting with additional medications is frequently necessary for better outcomes (Hollander *et al*., 2002; Pittenger and Bloch, 2014). A well-established augmentation strategy involves the addition of low-dose antipsychotic medications, with second-generation antipsychotics (in particular, risperidone 1–2 mg/day, and aripiprazole 10–15 mg/day), or first-generation antipsychotics (like haloperidol) representing the most effective agents (McDougle *et al*., 1994; Veale *et al*., 2014; Dold *et al*., 2015; Conti *et al*., 2024). Neurochemical disruptions, particularly in serotonergic (Insel *et al*., 1985), dopaminergic (Koo *et al*., 2010), and glutamatergic (Brennan *et al*., 2013) systems, are believed to contribute to OCD’s pathophysiology, thus providing the rationale for the use of antipsychotics in the treatment of this condition. Most first-generation and second-generation antipsychotics act by blocking postsynaptic dopamine D2 receptors, with some exceptions, including aripiprazole, cariprazine, and brexpiprazole, acting as partial D2 receptor agonists. Aripiprazole is often preferred for OCD augmentation due to its favorable side-effect profile. Its efficacy and tolerability as adjunctive therapy are supported by case reports, open-label studies (Pessina *et al*., 2009; Ak *et al*., 2011), two double-blind randomized trials (10–15 mg/day for 12–16 weeks) (Muscatello *et al*., 2011; Sayyah *et al*., 2012), and meta-analyses (Ducasse *et al*., 2014; Albert *et al*., 2017). Cariprazine, a partial agonist at dopamine D2/D3 receptors with higher D3 affinity, also targets serotonin 5-HT1A/5-HT2A receptors, but research on its use in OCD is limited: a case report described rapid OCD symptom remission in a schizophrenia patient after adding cariprazine to long-acting paliperidone (De Berardis *et al*., 2020), while a recent small retrospective study highlighted its potential effectiveness and safety as augmentation strategy in treatment-resistant OCD (Martiadis *et al*., 2024a).

Brexpiprazole is an atypical antipsychotic with effects of partial agonism at 5-HT1A, D2, and D3 receptors, and antagonism at 5-HT2A, 5-HT2B, 5-HT7, α1A, α1B, α1D, and α2C receptors. It is currently approved for the treatment of schizophrenia (including patients aged 13 years and older) (Greig, 2015), the adjunctive therapy of adult major depressive disorder (ibid.) and for agitation associated with Alzheimer’s disease-related dementia (Lee *et al*., 2023). A few clinical studies and case reports have explored its off-label use for other psychiatric conditions, with preliminary evidence suggesting potential efficacy, such as in the treatment of bipolar depression (Scala *et al*., 2023).

Compared to aripiprazole, it exhibits lower intrinsic agonist activity at the D2 receptor, implying a reduced propensity for D2 partial agonist-mediated side effects such as akathisia, insomnia, restlessness, and nausea. Partial agonism at D3 receptors could address negative symptoms and improve cognition and depressive features. Its partial agonism at 5-HT1A receptors may impart pro-cognitive, mood-enhancing, sedative, and anxiolytic effects. Additionally, 5-HT7 antagonism, in conjunction with 5-HT1A partial agonism, is believed to enhance cognitive and antidepressant effects, while also addressing negative symptoms of schizophrenia. Even modest 5-HT2C antagonism may further support its antidepressant action (Siwek *et al*., 2023).

In consideration of the abovementioned properties, along with clinical evidence supporting the efficacy of compounds with similar pharmacodynamic profiles, it is possible to hypothesize that brexpiprazole augmentation could constitute a safe and effective therapeutic strategy for the treatment of drug-resistant OCD. To the best of our knowledge, its potential utility in OCD has only been examined by a single recent retrospective study (Martiadis *et al*., 2024b). In this study, the authors collected data from 34 OCD-resistant patients who were given brexpiprazole (from 1 to 3 mg die) in augmentation to SRIs for at least 12 weeks; at the end of the study, 17 patients (50.0%) met the response criteria of ≥25% improvement in Yale-Brown Obsessive Compulsive Scale (Y-BOCS) total score vs baseline. To enhance current understanding of the topic, given the small sample size, we sought to contribute additional data from the experience at our center. Therefore, the aim of the present study was to further investigate the effectiveness and tolerability of augmentative brexpiprazole through a retrospective data collection from a real-world sample of patients with treatment-resistant OCD.

## Material and methods

### Participants

The present study retrospectively examined clinical data collected from outpatient services at the Fatebenefratelli-Sacco Mental Health Department in Milan, Italy. The recollection of clinical data started in May 2023 and ended in August 2024. Eligible participants were adults aged 18–65 years with a primary OCD diagnosis according to the Diagnostic and Statistical Manual of Mental Disorders, 5th edition criteria (APA, 2013). In case of psychiatric comorbidities, OCD had to be considered the primary disorder and directly responsible for the OC symptoms. To determine that OCD was a primary diagnosis, the clinicians carefully assessed the patients’ clinical history, focusing on the timeline of symptom onset. They prioritized the patients’ chief complaints, thoroughly evaluating the dominant symptoms and signs that caused the most functional impairment or distress. Inclusion criteria required a documented history of at least one trial of an SRI – including clomipramine, citalopram, escitalopram, fluoxetine, fluvoxamine, paroxetine, or sertraline – administered at therapeutic dosages and durations, according to international guidelines recommendations (Koran *et al*., 2007), with inadequate clinical response. Exclusion criteria included comorbid intellectual disability or substance/alcohol use disorders. Patients had to have received treatment with brexpiprazole for at least 12 weeks. Subjects who had additional changes to their concomitant psychopharmacological therapy, either at the beginning or during the trial with brexpiprazole, were also excluded.

### Materials

Symptom severity was assessed using the Y-BOCS (Goodman *et al*., 1989a; Pallanti, 2002), which consists of 19 items evaluating obsessions and compulsions. For the purposes of this study, only items 1–10 (excluding 1b and 6b) were used to calculate the Y-BOCS total score, which ranges from 0 to 40, providing a quantitative measure of OCD symptom severity. Subtotal scores for obsessions and compulsions were derived from items 1–5 (excluding 1b) and 6–10 (excluding 6b), respectively.

A treatment trial was considered unsuccessful if, after a minimum of 12 weeks, there was less than a 25% reduction in the Y-BOCS score (Goodman *et al*., 1989a; Pallanti, 2002). Written informed consent was obtained from all participants for the anonymous use of their clinical data in research or educational contexts. Additionally, written consent was acquired for any off-label treatments. Sociodemographic, clinical, and treatment variables were collected for each subject from medical records.

### Procedure

Participants received brexpiprazole at a dosage of 1–2 mg/day for a minimum of 12 weeks. Treatment was started at 1 mg/day for all patients, with subsequent dosage adjustments made according to the psychiatrist’s clinical assessment. The primary outcome measure was the change in Y-BOCS score between baseline (T0) and 12 weeks posttreatment initiation (T1), as this metric is widely used in meta-analyses to assess OCD treatment efficacy. Patients demonstrating a ≥25% reduction in total Y-BOCS score were classified as responders (Goodman *et al*., 1989b).

Adverse events, either reported by patients or observed by clinicians, were systematically recorded. Changes in Y-BOCS scores between baseline and week 12 were analyzed using paired *t*-tests and repeated measures analysis of variance (ANOVA). Data were anonymized and analyzed using SPSS version 27 (IBM SPSS Statistics for Windows, IBM Corp., Armonk, New York, USA). Significance was set at *P* < 0.05.

## Results

The initial cohort of patients with treatment-resistant OCD who started a treatment with brexpiprazole comprised 12 patients. Two subjects were subsequently excluded from the analysis: one male subject was diagnosed with comorbid bipolar disorder and did not receive SRI treatment, while the other was a male subject whose antidepressant regimen was modified following the initiation of brexpiprazole. Ten patients met the inclusion criteria and were deemed eligible for the study. Sociodemographic and clinical characteristics of each subject are presented in Table 1.

**Table 1:** Sociodemographic and clinical characteristics of the 10 patients included in the study

ID	Age (years)	Gender	Marital status	Employment	Obsessions type	Compulsion type	Insight	Tic-related	Psychiatric comorbidity	Age at onset (years)	Duration of illness (years)	DUI (months)	Current SRI	SRI dose	Brexpiprazole dose (mg)
1	65	M	Married	Retired	Aggressive	Miscellaneous	Poor	No	None	26	39	9	Clomipramine + Fluvoxamine	75 100	2
2	41	F	Married	Employed	Contamination	Cleaning/washing	Good	No	None	35	6	2	Sertraline	200	1
3	26	F	Single	Employed	Miscellaneous	Miscellaneous	Good	In the past	None	11	15	2	Sertraline	200	2
4	31	M	Single	Unemployed	Miscellaneous	Miscellaneous	Good	No	AD	25	6	48	Escitalopram	20	1
5	20	M	Single	Student	Miscellaneous	Checking	Poor	No	PD (cluster C)	18	2	4	Sertraline	150	1
6	35	F	Single	Employed	Somatic	Repeating rituals	Good	In the past	MDD	14	21	120	Sertraline	100	1
7	33	M	Single	Employed	Aggressive	Repeating rituals	Poor	In the past	None	26	7	6	Fluvoxamine	100	1
8	35	M	Single	Unemployed	Aggressive	Repeating rituals	Good	No	PD (cluster C)	15	20	60	Sertraline	200	2
9	23	M	Single	Unemployed	Miscellaneous	Miscellaneous	Good	No	PD (cluster A)	12	11	120	Fluvoxamine	300	2
10	63	M	Married	Employed	Religious	Miscellaneous	Good	No	MDD	15	48	60	Clomipramine	75	1

AD, anxiety disorder; DUI, duration of untreated illness; F, female; M, male; MDD, major depressive disorder; PD, personality disorder; SRI, serotonin reuptake inhibitor.

The mean age of the sample was 37.2 ± 15.4 years, with seven males and three females. The majority of them were single (70%) and a high percentage (30%) was unemployed. Mean age at OCD onset was 19.7 ± 7.9 years while mean illness duration was 17.5 ± 15.1 years. As previously indicated, patients with psychiatric comorbidities were not excluded, with the sample comprising two patients with a depressive disorder, one with an anxiety disorder, one with a cluster A personality disorder, and one with a cluster C personality disorder. Thirty percent of patients had a history of tics. Fifty percent had a family history of psychiatric disorders, all of which involved depressive disorders.

All patients took the drug continuously for at least 12 weeks. Six patients received the minimum dose of brexpiprazole (1 mg/day), while the remaining four received 2 mg/day. The SRI dosage remained unchanged throughout the augmentation period for all patients.

**Fig. 1:**
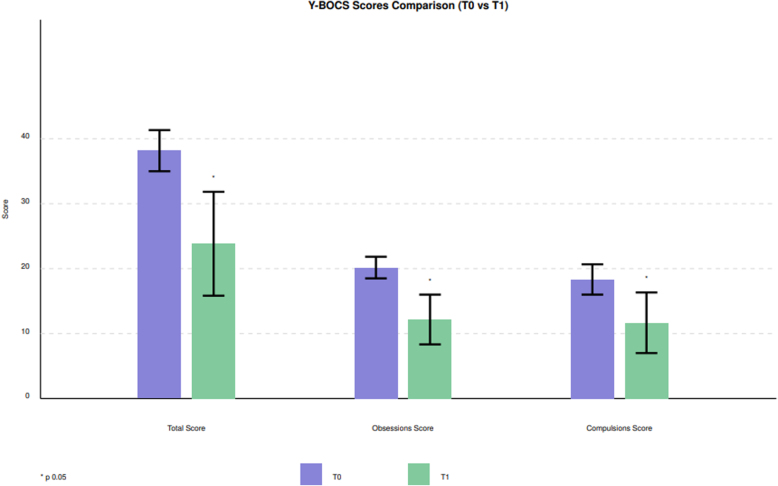
Comparison between Y-BOCS scores (total score and subscales) from T0 (baseline) to T1 (12 weeks). *Note*: Error bars represent the SD. Y-BOCS, Yale-Brown Obsessive Compulsive Scale.

Table 2 presents the changes in the total Y-BOCS score and its subscales from T0 to T1. Analysis of the Y-BOCS total score revealed a significant improvement at week 12 compared to baseline (paired *t*-test for mean Y-BOCS total score at week 12 vs baseline: *t* = 5.521, df = 9, *P* < 0.01). Significant improvements were observed in both the obsessions and compulsions subscales of the Y-BOCS at week 12 (paired *t*-test for the mean Y-BOCS obsession subscale: *t* = 7.201, df = 9, *P* < 0.01; paired *t*-test for the mean Y-BOCS compulsion subscale: *t* = 3.722, df = 9, *P* = 0.005) (see Fig. 1).

**Table 2:** Mean Y-BOCS scores (total score and subscales) from T0 (baseline) to T1 (12 weeks) and mean change

	Y-BOCS total score				Y-BOCS Obsessions score				Y-BOCS Compulsions score			
Subject	T0	T1	Change (%)	*P* value	T0	T1	Change (%)	*P* value	T0	T1	Change (%)	*P* value
1	26	17	34.62		15	8	46.67		11	9	18.18	
2	27	25	7.41		13	12	7.69		14	13	7.14	
3	33	25	24.24		17	12	29.41		16	13	18.75	
4	27	16	40.74		14	8	42.86		13	8	38.46	
5	32	21	34.38		16	11	31.25		16	10	37.50	
6	28	6	78.57		14	5	64.29		14	1	92.86	
7	28	18	35.71		14	7	50.00		14	11	21.43	
8	26	14	46.15		13	7	46.15		13	7	46.15	
9	27	25	7.41		16	14	12.50		11	11	0.00	
10	27	16	40.74		15	8	46.67		12	8	33.33	
Mean (SD)	28.1 (2.4)	18.3 (6)	35 (0.2)	0	14.7 (1.3)	9.2 (2.9)	37.7 (0.2)	0	13.4 (1.8)	9.1 (3.5)	31.4 (0.3)	0.05

A repeated-measures ANOVA was conducted to assess the effect of treatment over 12 weeks on the Y-BOCS total score. Mauchly’s test indicated a violation of the sphericity assumption, and thus, the degrees of freedom were adjusted using Huynh–Feldt estimates of sphericity. The analysis showed a significant effect (*F* = 30.478; df = 9.000; *P* < 0.001). Similar results were obtained for the respective subscales: Y-BOCS obsession subscale (*F* = 51.857; df = 9.000; *P* < 0.001) and Y-BOCS compulsion subscale (*F* = 13.856; df = 9.000; *P* = 0.005).

Qualitative analyses in terms of clinical response showed that, at the endpoint, seven patients (70%) demonstrated a ≥25% reduction in Y-BOCS total score from baseline, with five of them exhibiting a particularly strong clinical response (≥35% reduction). Adverse effects were mild, with one patient reporting mild sedation and another experiencing weight gain (<7.5% of baseline body weight). We found no statistically significant correlations between sociodemographic, clinical, or treatment variables and clinical response to brexpiprazole. Among nonresponders, the only shared characteristic was good insight. No sociodemographic or clinical variables were associated with nonresponse to brexpiprazole. At the end of the 12-week period, patients continued regular clinical and pharmacological monitoring with their respective caregivers, with any therapeutic adjustments made based on clinical judgment. To date, none of the patients has discontinued treatment with brexpiprazole. The periodic collection of psychometric data and side effects remains ongoing.

## Discussion

From a pharmacodynamic perspective, brexpiprazole exhibits a unique action profile, targeting both dopaminergic and serotonergic receptors, which may account for its potential efficacy in treating OCD.

To date, literature on the use of brexpiprazole as an augmentation strategy in patients with treatment-resistant OCD is scarce and very recent, with a single retrospective study on the topic (Martiadis *et al*., 2024b).

In our study, at the endpoint, the mean reduction in the Y-BOCS total score from T0 to T1 was 35%, and 70% of the overall sample met the response criterion. Notably, 50% of the whole sample and more than 70% of the responders (5 out of 7) showed a particularly robust response, with symptom reductions of ≥35%. This response rate seems to exceed that typically observed with other antidopaminergic agents, which are generally effective in about one-third of patients with treatment-resistant OCD (Dold *et al*., 2015; Grassi *et al*., 2021).

Specifically, risperidone, one of the most commonly used augmentation agents, has been shown in placebo-controlled studies to produce a Y-BOCS score reduction of approximately 20%. The small sample size and the descriptive design of our study prevent a direct comparison with the existing literature. Nonetheless, it is worth noting that the duration of the primary clinical trials is under 12 weeks, which may partially account for the diminished clinical response observed at the endpoint (McDougle *et al*., 2000; Hollander *et al*., 2003; Erzegovesi *et al*., 2005; Buchsbaum *et al*., 2006; Simpson *et al*., 2013).

As far as dopamine partial agonists are concerned, aripiprazole has demonstrated response rates (≥25% reduction in Y-BOCS score) ranging from 53 to 68.7% in previous randomized clinical trials with treatment-resistant OCD patients (Muscatello *et al*., 2011; Sayyah *et al*., 2012; Talaei *et al*., 2020). Cariprazine has shown a response rate of 61.5% as an augmentation to SRIs in treatment-resistant OCD in a recent small retrospective open study (Martiadis *et al*., 2024a), while another independent, recently published, open study with brexpiprazole, found 50% of patients achieving response at the 12 weeks endpoint (Martiadis *et al*., 2024b). Further larger studies are needed to confirm this data and gain better insight regarding the comparative effectiveness of the two compounds. The response rate observed in brexpiprazole-treated patients within our sample appears to be consistent with that reported for other dopamine partial agonists. The good response observed in our study may, in part, be attributed to its unique pharmacodynamic profile. Characterized by lower intrinsic agonist activity at the D2 receptor compared to other partial agonists, brexpiprazole likely exerts a more pronounced D2 antagonism, thereby modulating dopaminergic activity and improving therapeutic outcomes. Furthermore, brexpiprazole’s potential antidepressant and pro-cognitive properties could contribute to reducing the cognitive rigidity and distress typically associated with obsessions and compulsions.

In our study, brexpiprazole was well tolerated, with most adverse events being mild, transient, and not necessitating medical intervention. One patient reported mild sedation, which was managed by adjusting the dosing schedule to the evening. Sedation also was the most common side effect reported by patients in the study by Martiadis *et al*. (2024b). Another patient experienced weight gain, though this remained under 7.5% of baseline body weight. These results are consistent with the stronger antihistaminic activity of brexpiprazole (Bieńkowski, 2024). No patients discontinued treatment due to adverse effects. It is well known that partial agonism at D2 receptors can cause side effects, the most common being akathisia, nausea, and insomnia/arousal, constituting rather common adverse effects when using aripiprazole and cariprazine. Brexpiprazole exhibit a lower intrinsic agonist activity at the D2 receptor implying a reduced propensity for D2 partial agonist-mediated side effects (Stahl, 2016). Our study confirms the better acceptability and tolerability of brexpiprazole compared to aripiprazole and cariprazine, as documented in literature (Wang *et al*., 2023).

Several limitations must be acknowledged when interpreting these findings, including the small sample size, the retrospective open nature of the data, the lack of a mid-term evaluation and the absence of a control group. Additionally, due to the limited sample size, it was not feasible to conduct robust analyses to identify potential confounding variables. Undoubtedly, larger, double-blind, placebo-controlled trials are needed to further explore the efficacy of brexpiprazole. Future studies should also investigate the effects of higher doses or extended treatment durations, potentially assessing long-term outcomes. Notably, it would be valuable to examine whether combining brexpiprazole with specific selective SRIs might enhance its efficacy. Lastly, comparative studies between brexpiprazole and other augmentation strategies, particularly other atypical antipsychotics, would be highly informative.

Overall our results are promising, suggesting that brexpiprazole may offer a favorable effectiveness and tolerability profile in patients with treatment-resistant OCD.

## Acknowledgements

### Conflicts of interest

B.D. has received grant/research support from LivaNova, Inc., Angelini, and Lundbeck and Lecture Honoraria from Angelini, Janssen, Otsuka, Viatris, Boehringer, Bromatech, and Lundbeck. For the remaining authors, there are no conflicts of interest.
